# Cross-cultural adaptation and psychometric evaluation of the social frailty scale in Iranian older adults

**DOI:** 10.1186/s12877-024-04940-3

**Published:** 2024-04-24

**Authors:** Hanieh Zare, Zahra Tagharrobi, Mohammad Zare

**Affiliations:** Trauma Nursing Research Center, Kashan University of Medical Sciences, Kashan, Iran

**Keywords:** Frailty, Aged, Psychometrics

## Abstract

**Background:**

Social frailty is a holistic concept encompassing various social determinants of health. Considering its importance and impact on health-related outcomes in older adults, the present study was conducted to cross-culturally adapt and psychometrically evaluate the Social Frailty Scale in Iranian older adults in 2023.

**Methods:**

This was a methodological study. The translation and cross-cultural adaptation of the Social Frailty Scale 8-item (SFS-8) was conducted according to Wild’s guideline. Content and face validity were assessed using qualitative and quantitative methods. Then, 250 older adults covered by comprehensive health centers were selected using multistage random sampling. Participants completed the demographic questionnaire, the Abbreviated Mental Test score, the SFS-8, and the Lubben Social Network Scale. Construct validity was assessed by principal component analysis (PCA) and known-group comparisons. The Mann‒Whitney U test was used to compare social frailty scores between the isolated and non-isolated older adults. Internal consistency, equivalence, and stability were assessed using the Kuder-Richardson method, the intraclass correlation coefficient (ICC), the standard error of measurement (SEM), and the minimum detectable change (MDC). The ceiling and floor effects were also assessed. The data were analyzed using JASP 0.17.3.

**Results:**

The ratio and index of content validity and the modified kappa coefficient of all the items were 1.00. The impact score of the items was greater than 4.6. PCA identified the scale as a single component by removing two questions that could explain 52.9% of the total variance in the scale score. The Persian version of the Social Frailty Scale could distinguish between isolated and non-isolated older adults (*p* < 0.001). The Kuder–Richardson coefficient, ICC, SEM, and MDC were 0.606, 0.904, 0.129, and 0.358, respectively. The relative frequencies of the minimum and maximum scores obtained from the scale were 34.8 and 1.2, respectively.

**Conclusions:**

The Persian version of the Social Frailty Scale (P-SFS) can be used as a valid and reliable scale to assess social frailty in Iranian older adults.

## Background

Frailty is a health-threatening problem in older adults [1]. Frailty is an aging syndrome that reduces a person’s ability to resist stress and increases vulnerability to negative health outcomes [2, 3]. The global prevalence of frailty in older adults ranges from 4.0 to 59.1% [4]. In Iran, this rate is estimated to be between 10.4% and 33.43% [5, 6]. Frailty can occur due to adverse health outcomes, including physical conditions (e.g., heart failure), psychological conditions (e.g., cognitive impairment), and social conditions (e.g., isolation) [6]. The consequences of frailty in older adults include reduced motor function [7], falls [8], mental disorders [9], increased hospitalization rates [10], and death [11, 12]. Frailty is a multidimensional concept that encompasses physical, mental, and social dimensions [1]. Among these dimensions, social frailty is the least recognized and needs more attention from researchers [13].

Social frailty refers to a lack of social resources, social activities, and self-management abilities to meet people’s social needs [3, 14]. Bunt et al. (2017) state that social frailty is a continuum of being at risk of losing or having lost social resources, social behaviors, social activities, and self-management abilities to fulfill basic social needs [13]. Social frailty is significant for older adults due to significant social changes such as retirement, the death of a partner, changes in family structure, dependence on others, vague general policies, and the lack of specific national programs for older adults [3, 13]. In addition, social frailty has increased after the containment of the epidemic, the reduction of social activities, and the lack of social resources during the COVID-19 pandemic [15]. The global prevalence of social frailty ranges from 3.6 to 66.5% [16]. Therefore, the management of social frailty in older adults is critically important.

The assessment of social frailty helps to prevent and improve adverse health outcomes in frail older adults [15]. In recent years, several scales have been developed to assess social frailty [11, 14, 17–22]. However, no specific scale has yet been developed to assess social frailty in older adults in Iran. So far, general frailty scales have been used in Iranian studies, such as the Tilburg Frailty Indicator (TFI), which simultaneously measures all three frailty dimensions [2]. The use of non-specific social frailty scales has been criticized because these scales assess only the general concept of frailty with two or three questions, and the performance of these questions alone in terms of their ability to measure frailty is questionable and has been criticized [23].

A review of the social frailty scales revealed that some do not cover social activities [18]. Furthermore, despite the importance of social support in social frailty, more attention needs to be given to certain scales, such as getting help, having a relationship with another person, and respecting [11, 14, 17, 19, 20]. In addition, some scales are not suitable for older adults due to the large number of questions [21]. Due to the abovementioned limitations, the Social Frailty Scale 8-item (SFS-8) seems more suitable than other published scales [22].

The SFS-8 was developed by Pak et al. in Singapore in 2020 [22]. This scale was developed using the social production function (SPF) theory [13]. The SFS-8 assesses social frailty using eight items and three dimensions, including social resources, social activities and financial resources, and fulfillment of social needs [22]. The SFS-8 has been used in many studies, especially in Asian countries [12, 13, 24].

Considering the importance of social frailty in older adults, its negative health consequences, its impact on other dimensions of frailty, and the lack of a specific scale to assess social frailty in Iran, the present study was designed with the aim of cross-cultural adaptation and psychometric evaluation of the SFS-8 in older adults at Kashan in 2023. The introduction of a valid and reliable scale can help identify social frailty before it leads to physical, mental, and social problems. It can also reduce the negative burden of its consequences for older adults and society. In addition, an appropriate scale can help in planning effective strategies and policies for older adults.

## Methods

This study is a methodological study of translation and psychometric evaluation. The present study was designed and reported based on the COnsensus-based Standards for the selection of health Measurement INstruments (COSMIN) [25]. The study was conducted in five steps.

### Step 1: translation and cross-cultural adaptation

In this study, the translation guideline of Wild et al. [26] was used for translation and cross-cultural adaptation, including preparation, forward translation, reconciliation, back-translation, back-translation review, harmonization, cognitive debriefing, review of cognitive debriefing results and refinement, proofreading, and final reporting. The translators were independent of each other and experts in both languages and health topics. The original developers approved all the translation and cross-cultural adaptation processes of the SFS-8 scale.

### Step 2: content and face validity

As part of the content validity assessment, the initial Persian version of the SFS-8 was presented to ten gerontology, nursing, and psychology experts. The participants were asked to give their opinions on the simplicity, clarity, concept, scoring, and grammar of the items to ensure qualitative content validity. The quantitative content validity was assessed by the content validity ratio (CVR), the content validity index (CVI), and the modified kappa statistic. The interpretations of the CVR, CVI (I-CVI and S-CVI/Ave), and modified kappa statistic are based on Aryeh and Scully [27], Waltz et al. [28, 29], and Polit and Beck [28], respectively.

To assess face validity, a face-to-face interview was conducted with ten older adults (with differences in age, gender, and education). The older adults were asked to indicate their lack of understanding, difficulty level, and ambiguity of the items. To assess quantitative face validity, they rated the perceptibility of each item on a scale of 1 to 5 points; the impact score was calculated. An impact score of less than 1.5 indicates a comprehension problem [30].

### Step 3: data collection

The study population included all older adults who were covered by comprehensive health centers at Kashan in 2023. To achieve greater variation in the studied samples, at least 250 samples were considered. Some experts believe that the minimum sample size for a factor analysis with strict criteria is 250 [30, 31]. The inclusion criteria were Iranian citizenship, age 60 years and older, no cognitive disorders (based on the AMTs, a score above 7), no severe physical disorders such as Parkinson’s disease and stroke leading to dependence (based on the Integrated Health System (SIB) and self-reports), ability to communicate verbally, and consent to participate. Failure to complete the scales was considered an exclusion criterion.

The sampling method was a multi-stage sample. First, Kashan was divided into three presumptive socioeconomic regions; then, three comprehensive health centers were randomly selected from each region. The older adults were randomly selected according to the sample size and the number of older adults in each center. The random selection of the comprehensive health centers and the older adults in each center was based on a table of random numbers. The inclusion criteria of each selected older adult were assessed using the Integrated Health System (SIB) and self-reports. If they met the inclusion criteria and consented to participate in the study (by being called), they were invited to visit the centers to complete the scales. The timing of the visit to the center was coordinated with the opinions of the older adults. The older adults visited the center as part of their regular health care, and the data were collected via face-to-face interviews. If a selected older adult did not participate in the study, another sample was randomly replaced from the same center.

The data collection scales included [1] the demographic questionnaire [2], the Abbreviated Mental Test score (AMTs) [3], the SFS-8, and [4] the Lubben Social Network Scale (LSNS). The article’s first author completed all scales using an Integrated Health System (SIB) and interviews with older adults.

The demographic questionnaire included age, gender, marital status, number of children and siblings, educational level, native status, employment status, monthly income, type of housing, relocation status, insurance support status, smoking status, and underlying disease status. The qualitative content validity of this questionnaire was confirmed by ten faculty members in gerontology and nursing who were familiar with the psychometrics of the instrument.

The AMTs contains ten questions about important dates, times and years, famous people, occupations, place names, numerals, and addresses. The AMTs was scored between 0 and 10. In addition to confirming the face and content validity of the AMTs, the discriminant validity of the scale was also confirmed in various cognitive groups. The Cronbach’s alpha coefficient was calculated to be 0.76. In addition, the test-retest reliability was 0.89. The cut-off point of the Persian version of the AMTs was set at 7 (with a sensitivity of 94% and a specificity of 86%) [32]. The older adults completed the AMTs to investigate the presence of cognitive impairment prior to data collection.

The SFS-8 was developed by Pak et al. in Singapore in 2020. The SFS-8 assesses social frailty using eight items and three dimensions, including social resources (meeting with friends, need for advice and trust), social activities and financial resources (going out, eating and financial resources), and fulfilling social needs (loneliness and talking). Each item is scored as zero or one; the total score is estimated at 0 to 8. Based on the scores obtained, individuals were categorized into non-frail (score 0–1), pre-frail (score 3 − 2), and frail (score 4–8) groups. Experts have confirmed the face and content validity of the SFS-8. The exploratory factor analysis (EFA) revealed three factors that explained 50.5% of the total scale score. In addition, the SFS-8 can distinguish individuals with different states of depression, nutrition, and physical performance [22, 33]. In the present study, the SFS-8 was used for translation, cross-cultural adaptation, and psychometric assessment.

The LSNS was developed to assess the type, frequency, size, and closeness of older adults’ current social networks. This scale includes six items that are rated on a six-point Likert scale. The total score ranges from 0 to 30, so a higher score indicates a more robust social network and less social isolation. The cut-off point for LSNS is a score of less than 20, indicating a high risk of isolation. In addition to face and content validity, EFA led to the extraction of two dimensions (family and friends) from the Persian version of the LSNS. The confirmatory factor analysis confirmed the existence of this factor structure in the scale. The Cronbach’s alpha of the scale was calculated to be 0.896 [34, 35]. The LSNS was used for construct validity by known-group comparisons.

### Step 4: validity

After collecting the data, construct validity was assessed using PCA. The Kaiser–Meyer–Olkin (KMO) test and Bartlett’s test of sphericity were used to determine the scale’s ability to perform factor analysis. The extracted factors were considered using parallel analysis, eigenvalues (above one), and scree plot. According to the binary scoring (yes/no), PCA was performed using the polychoric correlation matrix. The minimum factor loading was set at 0.5.

The LSNS was used to assess construct validity by known-group comparisons. Social networks include various social connections and interactions that contribute to a person’s ability to maintain social resources, social activities, and self-management skills. It is essential for meeting basic social needs and avoiding social frailty. Therefore, it can be expected that an appropriate social frailty scale can differentiate between isolated and non-isolated people [34, 35]. All participants completed the LSNS with the Persian version of the Social Frailty Scale to assess this psychometric property. Then, the scores obtained with the Persian version of the Social Frailty Scale were compared based on the scores obtained and the cut-off point of the LSNS (scores less than 20 indicating a high risk of social isolation).

To assess ceiling and floor effects in the Persian version of the Social Frailty Scale, the relative frequency of samples that received the highest and lowest scores on this scale was investigated.

### Step 5: reliability

The internal consistency of the Persian version of the Social Frailty Scale was calculated using the Kuder–Richardson 20 (KR20). Twenty older adults completed the Persian version of the Social Frailty Scale after two weeks to assess stability using the test-retest method.

To assess the stability of the Persian version of the Social Frailty Scale, the standard error of measurement (SEM) was calculated using the formula SEM = SD × (1-ICC). The minimum detectable change (MDC) was also estimated using the formula SDC = 1.96×√2×SEM.

### Data analysis

All the statistical analyses were performed using JASP software (version 0.17.3). Kurtosis and skewness tests were used to assess the normality of the data (a range of ± 2 was considered indicative of a normal distribution). The Mann–Whitney U test and intraclass correlation coefficient (ICC) were used for known-group comparisons (based on LSNS) and test–retest scores, respectively. The significance level was less than 0.05 for all analyses.

### Ethics committee approval statement

The study was approved by the Ethics Committee of Kashan University of Medical Sciences and Health Services (Ethics Code: IR.KAUMS.NUHEPM.REC.1402.006). The aims, methods, advantages, and disadvantages of the study were explained and clarified in simple terms to all older adults. The participants were assured that all their information would be treated confidentially. It was also explained to the older adults that they could withdraw their cooperation at any time if they wished.

### Written and verbal informed consent statement

Written and verbal informed consent was obtained from all participants that approved by the Ethics Committee of Kashan University of Medical Sciences and Health Services. The cognitive status of all older adults was assessed before the study, and all were without cognitive impairment. If the older adults were illiterate or had a low level of education, informed consent was obtained in the presence of a witness; in addition, if necessary, a close family member was contacted to explain the study and its processes to them.

## Results

### Step 1: translation and cross-cultural adaptation

The draft Persian version of the SFS-8 was prepared with eight questions for psychometric evaluation. The answers to the scale questions were binary (yes/no).

### Step 2: content and face validity

Changes were made to items 2, 5, 6, 7, and 8 based on the content validity assessment. For example, the question “Are you facing a limitation in using your financial resources to pay for the medical services that need?” in item 8 was changed to “Do you have trouble paying for the medical services you need?“. In addition, the CVR, CVI (I-CVI and S-CVI), and modified kappa coefficient of the scale were 1.00. A slight change was made to the qualitative assessment of face validity to make item 6 easier to understand (adding examples for individuals and relatives). The impact score for all items was calculated to be greater than 4.6. Therefore, all the items were retained in this step [31].

### Step 3: data collection

In this study, 250 older adults were studied. All participants had an adequate cognitive score (based on the AMTs, a score of more than 7). The mean age of the participants was 66.092 years (± 6.269). Most participants were female, married, native to Kashan, had a primary education, were retired, had a monthly income between 5 and 10 million rials, had insurance support, and suffered from underlying diseases (Table 1).

**Table 1:** Participants’ demographic characteristics

Characteristics (*N* = 250)		N (%) Mean (± SD)
Age (Year)		66.092 (± 6.269)
Gender	Female	132 (52.8)
	Male	118 (47.2)
Marital Status	Single	8 (3.2)
	Married	208 (83.2)
	Widowed/Divorced/Separated	34 (13.6)
Number of Children		3.496 (± 1.597)
Number of Siblings		4.212 (± 2.339)
Educational Level	Illiterate	36 (14.4)
	Reading and Writing	29 (11.6)
	Elementary	75 (30.0)
	High School	37 (14.8)
	Diploma (Pre University)	55 (22.0)
	University	18 (7.2)
Native Status (Kashan Region)	Yes	229 (91.6)
	No	21 (8.4)
Employment	Housekeeper	115 (46.0)
	Retired	85 (34.0)
	Retired, but Working	22 (8.8)
	Employed	19 (7.6)
	Unemployed	9 (3.6)
Monthly Income	No Income	96 (38.4)
	Less than 5 million Rials	27 (10.8)
	5–10 million Rials	96 (38.4)
	More than 10 million Rials	31 (12.4)
Type of Housing	Owner of the House	231 (92.4)
	Renter	12 (4.8)
	Home of Relatives	7 (2.8)
Relocation (in the past year)	Yes	7 (2.8)
	No	243 (97.2)
Insurance Support	Yes	232 (92.8)
	No	18 (7.2)
Smoking Status	Cigarette	18 (7.2)
	Hookah	6 (2.4)
	Drugs	1 (0.4)
	None	225 (90.0)
Underlying Diseases	Yes	169 (67.6)
	No	81 (32.4)

### Step 4: validity

**Fig. 1:**
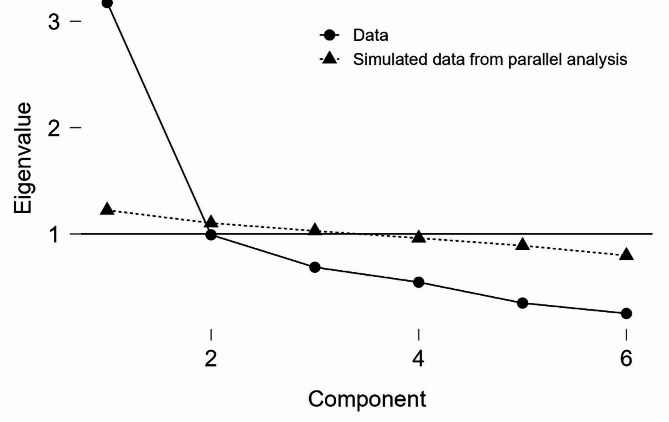
The scree plot of the SFS-8

The KMO statistic was calculated as 0.774 (items between 0.748 and 0.812). In addition, Bartlett’s test of sphericity was found to be significant (${\chi ^2}$ = 557.950, *p* < 0.001). Therefore, PCA was considered appropriate for identifying the structure of the factor model. PCA extracts a component based on parallel analysis with an eigenvalue greater than one. The scree plot also confirms the existence of a component in the scale. This factor explained 52.9% of the total variance in the scale score (Table 2; Fig. 1). Questions 1 and 8 were removed based on the analysis results (factor loadings and communities less than 0.3) and item analysis. Therefore, this scale was converted into a 6-item version.

**Table 2:** Principal component analysis and scores of the SFS-8 in older adults

No.	Items	Factor Loading*	Communality**	Persian version of the SFS-8		
				Yes N (%)	No N (%)	Mean (± SD)
1	Do you live alone?	Was removed				
2	Do you go out less frequently compared with last year?	0.595	0.354	111 (44.4)	139 (55.6)	0.444 (± 0.498)
3	Do you occasionally meet your friends?	0.620	0.384	70 (28.0)	180 (72.0)	0.280 (± 0.450)
4	Do you talk with someone every day?	0.759	0.576	236 (94.4)	14 (5.6)	0.056 (± 0.230)
5	Do you turn to family or friends for advice?	0.742	0.550	206 (82.4)	44 (17.6)	0.176 (± 0.382)
6	Do you spend at least one meal in a day with another person (such as spouse, child, or friends)?	0.881	0.775	220 (88.0)	30 (12.0)	0.120 (± 0.326)
7	Do you have someone to confide in?	0.732	0.536	208 (83.2)	42 (16.8)	0.168 (± 0.375)
8	Are you facing a limitation to use your financial resources to pay for the medical services that need?	Was removed				
Eigenvalue (6-item)				3.176		
The variance explained (%)				52.9		
The total score of Persian version of the social frailty scale***				1.244 (± 1.345)		

* The minimum factor loading was 0.5 (lower factor loadings are not included)

** The minimum commonality was set at 0.3 (lower commonalities are not included)

*** The total score was calculated in the range of 0 to 6. Therefore, for each sample, the answer “yes” for question 1 and “no” for questions 2 to 6 were considered one score and the other answers as zero. The total score was then calculated. A higher score means greater vulnerability

Based on the LSNS, 120 and 130 individuals were categorized as isolated and non-isolated, respectively. The social frailty scores were estimated to be 1.675 (± 1.456) and 0.846 (± 1.096) for the isolated and non-isolated older adults, respectively. The difference between the two groups was statistically significant (Z = 4925.00, *p* < 0.001).

The relative frequencies of the minimum and maximum possible scores of the Persian version of the Social Frailty Scale were 34.8 and 1.2, respectively.

### Step 5: reliability

The internal consistency of the Persian version of the Social Frailty Scale was calculated to be 0.606. The ICC average measure was calculated as 0.904 between the results of the two test-retest evaluations using the two-way random model of absolute agreement. The ICC was estimated with a 95% confidence interval between 0.762 and 0.962, which was statistically significant (*p* < 0.001).

The SEM of the scale was calculated as ± 0.129 and estimated with a 95% confidence interval in the population range of 0.124 to 0.382. In addition, the MDC was reported to be 0.358.

## Discussion

The aim of the present study was the cross-cultural adaptation and psychometric evaluation of the Social Frailty Scale in older adults at Kashan in 2023.

Sousa et al. (2011) believe that if the research team’s semantic, terminological, experimental, and perceptual equalities are respected in the translation process, it can be claimed that the scale has been translated by the principles of cross-cultural adaptation [36]. For quantitative content and face validity, changes were made for simplicity, clarity, and understanding of concepts. Regarding the CVR, CVI, modified kappa statistic, and impact score, it can be claimed that the Persian version of the SFS-8 scale has the necessary criteria to verify content and face validity.

According to PCA, two items had very low factor loading and commonality (less than 0.3); therefore, these two items were removed, including No. 1 (Do you live alone? ) and No 0.8 (Are you facing a limitation in using your financial resources to pay for the medical services that need? ). One of the possible reasons is the characteristics of the participants. The variables “marital status” and “insurance support” showed less dispersion in the samples, which affected two related items in the scale. However, the concept of deleted item No. 1 is closely associated with items No. 4 (Do you talk with someone every day? ) and No. 6 (Do you spend at least one meal in a day with another person? ); therefore, these items can cover its meaning. Moreover, the culture of Iranian society is such that most older adults live with their families, and care is mainly provided at home, which is very common at Kashan. Therefore, the importance of this item for assessing the social frailty of Iranian older adults is lower than that of other concepts. Pek et al. (2020) also showed that items No. 1 and No. 4 are one factor and have a high correlation [22]. To remove item No. 8, most Iranians benefited from comprehensive insurance coverage, and the primary health care (PHC) program was widely used. Therefore, it seems that older adults face fewer challenges related to this concept, which is likely to have less impact on social frailty among older adults in Iran.

According to the PCA, one component was extracted that explained 52.9% of the total variance in the scale score. In this regard, Reio and Shuck (2015) state that achieving at least 50% of the scale score is an acceptable criterion in factor analysis [37]; therefore, the variance calculated for the single-factor and 6-item structure of the Social Frailty Scale appears to be acceptable. These results are consistent with those of Pak et al. (2020); in their study, three factors were extracted from the 8-item scale of the SFS-8, which explained 50.5% of the total variance of the scale. However, their results appear to be overestimated. Although it is a binary scale (yes/no), Pak et al. (2020) used the Pearson correlation matrix [22], which makes the results of the study questionable.

The validity results of the known-group comparisons showed that the Persian version of the Social Frailty Scale can significantly differentiate between isolated and non-isolated older adults (based on the LSNS). Increased social participation, including frequent communication and participation in social activities, can reduce the risk of frailty in middle-aged and older adults. Thus, maintaining a robust social network helps to reduce social frailty and promote health and quality of life [34, 35]. In addition to social isolation, social frailty is associated with other concepts due to its multidimensional nature. For example, in the study of Pak et al. (2020), the SFS-8 was able to predict depression significantly. Due to the similarity of many social concepts, such as family structure and social interactions, the present study has been aligned with other studies, and the Social Frailty Scale has high discriminant value among social concepts [37].

The relative frequencies of the minimum and maximum scores of the Persian version of the Social Frailty Scale were 34.8% and 1.2%, respectively. Although the scale does not have a ceiling effect, the results showed a floor effect (approximately 35%). This effect may limit the ability to discriminate between participants with low scores and may not accurately represent the actual score of the concept under study in this group [31, 38]. The possible cause of the floor effect in the scale is the selection of samples from society and the greater cooperation of physically, mentally, and mentally healthy older adults. In addition, some factors can cause older adults to give socially desirable answers to questions, such as being embarrassed or trying to make the situation look normal.

The reliability coefficient of the 6-item version of the scale was acceptable [39], which confirms its internal consistency; however, another study did not examine the internal consistency of the SFS-8 scale. The coefficient of agreement between the test-retest scores of the Persian version of the Social Frailty Scale was calculated at an acceptable level. Koo and Li (2016) considered correlation coefficients of at least 0.6 to be acceptable [40]; therefore, this scale has good test-retest reliability.

The SEM was used to assess absolute reliability. The results showed that the score can change by ± 0.129 when the older adult scale is completed again. Due to the scale’s scoring range and binary scoring, the calculated SEM was considered small, indicating the scale’s desired stability [31]. The MDC was calculated as 0.358. The MDC showed the smallest change in score that can be perceived by patients, caregivers, or researchers. Therefore, this index should be considered an important criterion when evaluating the accuracy and clinical relevance of a scale [39]. The calculated MDC is influenced by the concept of social frailty, which is considered desirable.

The study participants showed considerable diversity in terms of age, gender, education level, employment, monthly income, and underlying health conditions. This diversity is considered a strength of our research as it increases the generalizability of the results. However, some variables, such as being native, marital status, and insurance support, were less diverse, which is a limitation of our study that may have affected the results. Another limitation of the study is that there may be a small number of older adults who are not covered by comprehensive health centers, making it impossible to sample them. Moreover, the greater collaboration of older adults with lower levels of frailty was found to be a limitation of this study. Additionally, confirmatory factor analysis (CFA) was not performed due to the difficulty of sampling and the time limitation of the research project, which is a master’s thesis. Future research should use CFA techniques with a larger sample size to verify the proposed factor structures and evaluate model fit indices. This will help in establishing stronger psychometric properties for the proposed scale.

It is recommended that a diagnostic study be conducted to determine the cut-off point for the Persian version of the Social Frailty Scale. It was suggested that the two deleted items be considered when using the scale with non-Iranian older adults and in different contexts. Considering the SDC and the floor effect in this study, it is recommended that the psychometric properties of the Social Frailty Scale be investigated in future studies with Likert scoring and through the participation of socially frail older adults. It appears that assessing the scale’s psychometric properties with a Likert response can also reduce social desirability bias in the samples.

## Conclusions

Following the translation and cross-cultural adaptation of the SFS-8 according to the guideline of Wild et al., the content and face validity of the scale were assessed and confirmed using both quantitative and qualitative methods. Construct validity using the PCA method revealed that the scale was a single factor explaining 52.9% of the total variance. The Persian version of the scale can differentiate between isolated and non-isolated older adults. In addition, this scale has good reliability. The Persian version of the scale has a floor effect; however, this scale can identify frail older adults, especially those with risk factors for frailty. Finally, the Persian version of the Social Frailty Scale (P-SFS) with six items was developed, which has desirable psychometric properties.

## Data Availability

The datasets generated and/or analyzed during the current study are not publicly available due to the confidentiality of the information of the participants, but are available from the corresponding author on reasonable request.

## Abbreviations

AMTs: Abbreviated Mental Test scale
COSMIN: COnsensus-based Standards for the selection of health Measurement Instruments
CVI: Content Validity Index
CVR: Content Validity Ratio
EFA: Exploratory Factor Analysis
ICC: Intraclass Correlation Coefficient
I-CVI: Item-Content Validity Index
KMO: Kaiser–Meyer–Olkin
KR20: Kuder–Richardson 20
LSNS: Lubben Social Network Scale
MDC: Minimum Detectable Change
PCA: Principal Component Analysis
PHC: Primary Health Care
P-SFS: Persian Social Frailty Scale
S-CVI/Ave: Scale-Content Validity Index/Average
SEM: Standard Error of Measurement
SFS-8: Social Frailty Scale 8-item
SPF: Social Production Functions
TFI: Tilburg Frailty Indicator
